# Kinome Expansion in the Fusarium oxysporum Species Complex Driven by Accessory Chromosomes

**DOI:** 10.1128/mSphere.00231-18

**Published:** 2018-06-13

**Authors:** Gregory A. DeIulio, Li Guo, Yong Zhang, Jonathan M. Goldberg, H. Corby Kistler, Li-Jun Ma

**Affiliations:** a Department of Biochemistry and Molecular Biology, University of Massachusetts Amherst, Amherst, Massachusetts, USA; b The Broad Institute of MIT and Harvard, Cambridge, Massachusetts, USA; c USDA ARS, Cereal Disease Laboratory, St. Paul, Minnesota, USA; Carnegie Mellon University

**Keywords:** *Fusarium oxysporum* species complex, TOR kinase, accessory chromosome, histidine kinase, kinome

## Abstract

The Fusarium oxysporum species complex (FOSC) is a group of soilborne pathogens causing severe disease in more than 100 plant hosts, while individual strains exhibit strong host specificity. Both chromosome transfer and comparative genomics experiments have demonstrated that lineage-specific (LS) chromosomes contribute to the host-specific pathogenicity. However, little is known about the functional importance of genes encoded in these LS chromosomes. Focusing on signaling transduction, this study compared the kinomes of 12 F. oxysporum isolates, including both plant and human pathogens and 1 nonpathogenic biocontrol strain, with 7 additional publicly available ascomycete genomes. Overall, F. oxysporum kinomes are the largest, facilitated in part by the acquisitions of the LS chromosomes. The comparative study identified 99 kinases that are present in almost all examined fungal genomes, forming the core signaling network of ascomycete fungi. Compared to the conserved ascomycete kinome, the expansion of the F. oxysporum kinome occurs in several kinase families such as histidine kinases that are involved in environmental signal sensing and target of rapamycin (TOR) kinase that mediates cellular responses. Comparative kinome analysis suggests a convergent evolution that shapes individual F. oxysporum isolates with an enhanced and unique capacity for environmental perception and associated downstream responses.

**IMPORTANCE** Isolates of Fusarium oxysporum are adapted to survive a wide range of host and nonhost conditions. In addition, F. oxysporum was recently recognized as the top emerging opportunistic fungal pathogen infecting immunocompromised humans. The sensory and response networks of these fungi undoubtedly play a fundamental role in establishing the adaptability of this group. We have examined the kinomes of 12 F. oxysporum isolates and highlighted kinase families that distinguish F. oxysporum from other fungi, as well as different isolates from one another. The amplification of kinases involved in environmental signal relay and regulating downstream cellular responses clearly sets *Fusarium* apart from other *Ascomycetes*. Although the functions of many of these kinases are still unclear, their specific proliferation highlights them as a result of the evolutionary forces that have shaped this species complex and clearly marks them as targets for exploitation in order to combat disease.

## Abstract (precis)

Isolates of Fusarium oxysporum are adapted to survive a wide range of host and nonhost conditions. In addition, F. oxysporum was recently recognized as the top emerging opportunistic fungal pathogen infecting immunocompromised humans. The sensory and response networks of these fungi undoubtedly play a fundamental role in establishing the adaptability of this group. We have examined the kinomes of 12 F. oxysporum isolates and highlighted kinase families that distinguish F. oxysporum from other fungi, as well as different isolates from one another. The amplification of kinases involved in environmental signal relay and regulating downstream cellular responses clearly sets *Fusarium* apart from other *Ascomycetes*. Although the functions of many of these kinases are still unclear, their specific proliferation highlights them as a result of the evolutionary forces that have shaped this species complex and clearly marks them as targets for exploitation in order to combat disease.

## INTRODUCTION

A constantly evolving genome provides the genetic foundation for an organism to adapt to challenging environments. The Fusarium oxysporum species complex (FOSC) represents an exceptional model to study the relationship between genome evolution and organism adaptation. Phylogenetically related members within the F. oxysporum species complex include both plant and human pathogens and are collectively capable of causing plant wilt diseases in more than 100 plant species. Individual isolates often exhibit a high degree of host specificity, reflecting rapid adaptation to particular host environments in a very short evolutionary time frame (less than 30 million years ago [Mya]) (1). *Forma specialis* has been used to describe strains that are adapted to a specific host. Comparative studies revealed that horizontally acquired lineage-specific (LS) chromosomes contribute to the host-specific pathogenicity of each *forma specialis* (2–4).

Protein kinases (which we refer to as kinases herein for simplicity) are key regulators within cellular regulatory networks. They transduce extracellular and intracellular signals by modifying the activity of other proteins, including transcription factors, enzymes, and other kinases via phosphorylation (5–7). A “protein kinome” encompasses all protein kinases within a genome (8–13) and can be divided into several families, including the STE (homologs of the yeast sterile kinases), CK1 (casein kinase 1), CAMK (Ca^2+^/calmodulin-dependent protein kinase), CMGC (cyclin-dependent kinases [CDKs]), AGC (protein kinase A, G, and C families), HisK (histidine kinase), Other, and Atypical families (14). Some fungal kinomes have been functionally characterized (15–21).

To study the variation and evolution of kinases with respect to FOSC host-specific adaptation, we compared the kinomes of 12 Fusarium oxysporum isolates, including 10 plant pathogens, 1 human-pathogenic strain, and 1 nonpathogenic biocontrol strain. In addition, we have included seven ascomycete fungal genomes available in public domains. Our study revealed a clear correlation between the genome size and the size of the kinome of an organism. Due in part to the acquisition of LS chromosomes, the sizes of FOSC genomes are larger than other genomes included in this study, and so are their kinomes. Regardless of kinome size, we observed a highly conserved kinome core of 99 kinases among all fungal genomes examined. In contrast to this remarkably stable core, variation among families and subfamilies was observed across levels of taxonomic classification. Most interestingly, we observed the expansion of the target of rapamycin (TOR) kinase and histidine kinases among FOSC genomes. Monitoring nutrient availability and integrating intracellular and extracellular signals, the TOR kinase and its associated complex serve as a central regulator of cell cycle, growth, proliferation, and survival (22). Increasing the copy number of certain kinases may enable new functions or add new temporal variations to existing pathways. The repeated, but independent, expansion of certain kinase families among FOSC genomes may suggest a fine-tuning of similar pathways in responding to different host defenses or abiotic environmental challenges.

## RESULTS

### Overall kinase conservation defines a core kinome among ascomycete fungi.

**FIG 1:**
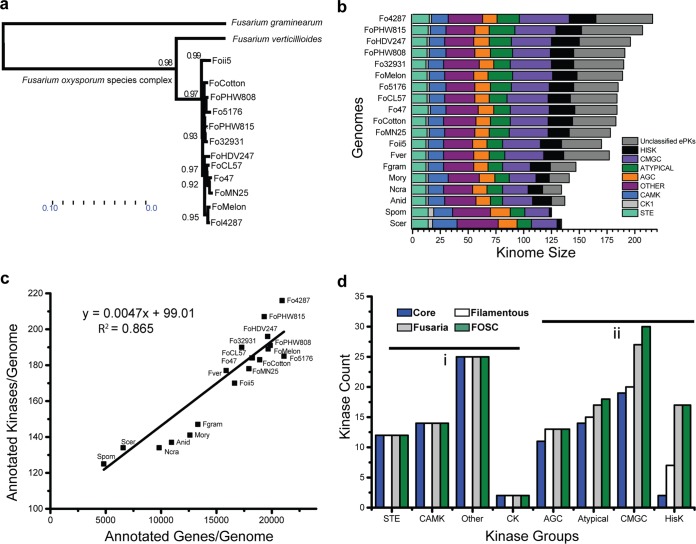
Kinomes across *Ascomycota*. (a) A neighbor-joining tree constructed from conserved genome genes showing the phylogenetic relationship among the *Fusarium* species used in this study. (b) Kinases were broken up into major families and abundance per family plotted per species. The individual groups are indicated by colors as shown in the color key. (c) Total gene count plotted against kinome size in each genome. The origin coordinates are *y* = 100 and *x* = 2,500. The shortened genome names used in panels a to c are given in Table 1. (d) By eliminating kinases missing from more than one species, we compiled “conserved kinomes” for *Ascomycetes* (all fungi here), filamentous fungi (all but S. cerevisiae and S. pombe), the genus *Fusarium*, and the FOSC. Some families remain relatively stable across species (group i [AGC, CAMK, CK, Other, and STE]), while others expand due to increased copy number or increased subfamily number (group ii [Atypical, CMGC, and HisK]). The total sizes of conserved kinomes follow: 99 for ascomycetes, 108 for filamentous fungi, 126 for fusaria, and 128 for FOSC.

We compared 19 ascomycete fungal genomes (Table 1), including 12 strains within the Fusarium oxysporum species complex (FOSC), two sister species close to F. oxysporum (Fusarium graminearum and Fusarium verticillioides) (Fig. 1a), two yeast genomes (Saccharomyces cerevisiae and Schizosaccharomyces pombe), two model fungal species (Neurospora crassa and Aspergillus nidulans), and an additional plant pathogen, Magnaporthe oryzae. With the exception of the two yeasts, other genomes were annotated at the Broad Institute using the same genomic annotation pipeline (23). All kinomes were predicted using the kinome prediction pipeline (24) with the same parameters. Kinases were classified by Kinannote into the STE, CK1, CAMK, CMGC, AGC, HisK, Other, and Atypical families (Fig. 1b; see Table S1 in the supplemental material). Kinases that could not be classified within specified statistical parameters were categorized as unclassified.

10.1128/mSphere.00231-18.6 TABLE S1  Gene IDs of predicted kinomes. Kinomes predicted by Kinannote are displayed by gene ID. Columns represent the kinases within a single species, while rows represent the various families of kinase as predicted by Kinannote. If a single family contained more than one kinase, all gene IDs are listed within that cell. Download TABLE S1, XLSX file, 0.6 MB. Copyright © 2018 DeIulio et al. 2018 DeIulio et al. This content is distributed under the terms of the Creative Commons Attribution 4.0 International license.

**TABLE 1:** Fungal genomes used in this study

Fungal strain or species (abbreviation^a^)	Strain ID^b^	Genome size (MB)^c^	Total no. of genes	Kinome size (no. of genes)	Host	NCBI accession no.
F. oxysporum f. sp. lycopersici 4287 (Fo4287)	NRRL 34936	61.35	20,925	216	Tomato	GCA_000149955.2
F. oxysporum Fo47 (Fo47)	NRRL 54002	49.66	18,191	184		GCA_000271705.2
F. oxysporum Fo5176 (Fo5176)	NRRL 66176	54.94	21,087	185	*Arabidopsis*	GCA_000222805.1
F. oxysporum f. sp. radicis-lycopersici (FoCL57)	NRRL 26381	49.35	18,238	184	Tomato	GCA_000260155.3
F. oxysporum f. sp. vasinfectum (FoCotton)	NRRL 25433	52.91	18,905	183	Cotton	GCA_000260175.2
F. oxysporum HDV247 (FoHDV247)	NRRL 54007	55.18	19,623	196	Pea	GCA_000260075.2
F. oxysporum f. sp. cubense (Foii5)	NRRL 54006	46.55	16,634	170	Banana	GCA_000260195.2
F. oxysporum f. sp. melonis (FoMelon)	NRRL 26406	54.03	19,661	189	Melon	GCA_000260495.2
F. oxysporum f. sp. lycopersici MN25 (FoMN25)	NRRL 54003	48.63	17,931	178	Tomato	GCA_000259975.2
F. oxysporum f. sp. conglutinans (FoPHW808)	NRRL 54008	55.57	19,854	191	Brassica	GCA_000260215.2
F. oxysporum f. sp. raphani (FoPHW815)	NRRL 54005	53.49	19,306	207	Brassica	GCA_000260235.2
F. oxysporum 32931 (Fo32931)	NRRL 32931	47.90	17,280	190	Human	GCA_000271745.2
F. graminearum PH-1 (Fgram)	NRRL 31084	36.44	13,321	147	Wheat	GCA_000240135.3
F. verticillioides 7600 (Fvert)	NRRL 20956	41.77	15,869	177	Corn	GCA_000149555.1
Magnaporthe oryzae 70-15 (Mory)	FGSC 8958	41.02	12,593	141	Rice	GCA_000002495.2
Neurospora crassa OR74A (Ncra)	FGSC 987	41.03	9,820	134		GCA_000182925.2
Aspergillus nidulans FGSC A4 (Anid)	NRRL 194	30.06	10,937	147		GCA_000149205.2
Saccharomyces cerevisiae S288C (Scer)	ATCC 204508	12.07	6,572	134		GCA_000146045.2
Schizosaccharomyces pombe (Spom)	ATCC 24843	12.57	4,820	125		GCA_000002945.2

a The shortened fungal name used in Fig. 1.

b NRRL, Agricultural Research Service Culture Collection; FGSC, Fungal Genetic Strain Collection; ATCC, American Type Culture Collection.

c MB, megabases.

Overall, there is a positive correlation between the total number of proteins encoded in a genome (*y*) and the total number of protein kinases within that genome (*x*) (*y* = 0.00473*x* + 99.01; *R*^2^ = 0.86) (Fig. 1c). The dependency of these two variables (number of kinases versus number of proteins) also points to a potential minimal number of 99 kinases for each ascomycete genome. Independently, we identified a core ascomycete kinome based on sequence conservation, taking a conservative approach by excluding kinase subfamilies that were missing from more than a single species. This conserved core kinome contains genes encoding kinases from all major kinase families, including 12 kinases in the STE family, 2 kinases in the CK1 family, 14 kinases in the CAMK family, 11 kinases in the AGC family, 19 kinases in the CMGC family, 2 kinases in the HisK family, 14 kinases in the Atypical family, and 25 kinases in the Other family (Fig. 1d), totaling 99 conserved kinase orthologs, in agreement with the prediction of the regression model. In addition to the ascomycete core kinome, we also compiled conserved kinomes for filamentous fungi (excluding both S. cerevisiae and S. pombe), the genus *Fusarium*, and the FOSC, each containing 108, 127, and 131 kinases, respectively. Interestingly, the sizes of the STE, CK1, CAMK, and Other families remain effectively constant across phylogenetic divisions, while AGC, Atypical, CGMC, and HisK families exhibit trends of continuous expansion, illustrated as categories i and ii in Fig. 1d.

### (i) The category I (constant group) includes STE, CK, CAMK, and Other families. *(a) STE family*.

The STE kinases transduce diverse signals, including osmolality, pheromone recognition, and cell wall integrity, and alter cell growth patterns in response to extracellular changes (25). The STE family includes mitogen-activated protein (MAP) kinase kinase (MAPKK), MAP kinase kinase kinase (MAPKKK), and their upstream activators that function in MAPK signaling cascades. Evolutionarily conserved, a MAPKKK phosphorylates a MAPKK, and activated MAPKK phosphorylates a MAPK; activated MAPKs, belonging to the CMGC family described below, phosphorylate other proteins to further control gene expression and cellular function. The 12 conserved fungal STE kinases are divided into nine subfamilies with a single kinase each, except the STE/PAKA and STE/YSK subfamilies that have two and three kinases, respectively (Table S2). Except S. cerevisiae, which lacks a single STE/YSK ortholog, all other genomes contain 12 orthologs. Limited copy number variation of the STE family confirms the functional importance of these signaling pathways across *Ascomycota*.

10.1128/mSphere.00231-18.7 TABLE S2  Conserved kinase counts. The number of kinases found belonging to each class within each species is listed, with the species shown in columns and kinase families shown in rows. The number of kinases within each family is color coded from yellow to dark green with green indicating higher copy number. An “x” under the appropriate column at the right indicates if that kinase family was conserved among all ascomycetes, filamentous ascomycetes, the genus *Fusarium*, or only the Fusarium oxysporum species complex. Notes on the left indicate the individual classes each histidine kinase belonged to. The total kinase count is listed at the bottom below each species' column. Download TABLE S2, XLSX file, 0.02 MB. Copyright © 2018 DeIulio et al. 2018 DeIulio et al. This content is distributed under the terms of the Creative Commons Attribution 4.0 International license.

### *(b) CK and CAMK families*.

Like the STE kinases, the CK and CAMK kinases are highly conserved among the ascomycete fungal genomes. Each genome has genes encoding 2 members of the CK1 conserved subfamily and 14 members of the CAMK conserved subfamily. All filamentous fungal genomes have a single copy of a gene in each subfamily. The S. pombe genome lacks an ortholog of both the CAMK/CMK and the CAMK/Rad53 kinases, while the S. cerevisiae genome lacks an ortholog of the CAMK/CAMK1 kinase. An ancient kinase family of serine/threonine-selective enzymes, CK1 kinases are present in most eukaryotic organisms and are involved in important signal transduction pathways, including regulating DNA replication and the circadian rhythm. In S. cerevisiae, the two CK1 kinases function in morphogenesis, proper septin assembly, endocytic trafficking, and glucose sensing (26). Found in almost all eukaryotic cells, CAMK kinases are activated by Ca^2+^ fluctuations within the cell and control tip growth, branching, spore production, cell cycle progression, and secretion, among others (27).

### *(c) Other kinase family*.

The Other kinase family includes several unique eukaryotic protein kinases (ePKs) that cannot be placed into any of the major ePK groups based on sequence similarity. There are 25 kinases in the Other kinase family, which is further divided into 24 subfamilies. Except for the Other/CAMKK subfamily, which contains two kinases, each subfamily of the Other kinase family has a single copy within each genome. The Other kinases account for a quarter of the ascomycete core kinome, and many kinases within this group are involved in basal cellular functions, including cell cycle control (28–30).

### (ii) The category II (variable group) contains AGC, Atypical, CMGC, and histidine kinases (HisKs). *(a) AGC family*.

The AGC family contains 11 subfamilies with a single orthologous copy each. All subfamilies are present in all genomes, except the AGC/YANK kinase is absent from the S. cerevisiae genome. AGC family kinases are cytoplasmic serine/threonine kinases regulated by secondary messengers such as cyclic AMP (protein kinase A [PKA]) or lipids (protein kinase C [PKC]) (31). They are involved in signaling pathways that orchestrate growth and morphogenesis, as well as response to nutrient limitation and other environmental stresses (32).

### *(b) Atypical kinase family*.

There are 11 kinases in the Atypical kinase family, which is further divided into 10 subfamilies with a single copy in each subfamily, except the Atypical/ABC-1B subfamily which has two. Distinctively, kinases of the Atypical family lack the canonical ePK domain, but they have protein kinase activity (13). This family also includes many functionally important kinases, including the TOR kinase, a major regulatory hub within the cell controlling nutrient sensing, cell cycle progression, stress responses, protein biosynthesis, and various mitochondrial functions (33). Other subfamilies of this group also play significant roles in cell cycle progression (RIO [*ri*ght *o*pen reading frame]), mRNA degradation (PAN), and the DNA damage response (ATM [ataxia-telangiectasia mutated], ATR [ataxia-telangiectasia and Rad3-related], and TRRAP [transformation/transcription domain-associated protein]).

### *(c) CMGC family*.

The CMGC family is an essential and large group of kinases found in all eukaryotes, accounting for roughly 20% of most kinomes. The group is comprised of diverse subfamilies that control cell cycle, transcription, as well as kinases involved in splicing and metabolic control (34), including cyclin-dependent kinases (CDKs), mitogen-activated protein kinases (MAP kinases), glycogen synthase kinases (GSKs), serine/arginine protein kinase (SRPK), and CDK-like kinases. Mitogen-activated protein kinases (MAPKs) form the last step in the three-step MAPK signaling cascades, which regulate functions from mating and invasive growth, to osmosensing and cell wall integrity (35). CDKs are widely known as controllers of the cell cycle and transcription (34). Kinannote reported 23 subfamilies of CMGC kinase, of which 18 were conserved and included in the conserved kinome. Each subfamily contains a single kinase, except for the CMGC/ERK1 subfamily that contributes two. The S. cerevisiae kinome lacks 3 out of the 18 conserved subfamilies (CMGC/CDK11, CMGC/DYRK2, CMGC/PRP4).

### *(d) HisK family*.

Differing from other kinase groups discussed above, the HisK family is widely distributed throughout prokaryotes and eukaryotes outside the metazoans. This group of kinases sense and transduce many intra- and extracellular signals (36–39). Distinctively, this family has the most significant expansion from yeast to filamentous fungi and is further expanded in the FOSC genomes (Fig. 1d). All HisKs are classified into 11 families, or classes, based on the conservation of the H-box domain (36). However, only two classes (class V and class X) can be considered orthologous among most ascomycete genomes, present in all genomes here except S. cerevisiae (Table S2). The class V HisK is orthologous to the S. pombe Mak1, while the single conserved class X kinase is orthologous to the S. pombe Mak2/Mak3 kinases. Mak2 and Mak3 are known peroxide sensors (40), while all Mak1/2/3 kinases are predicted to have a central role controlling the stress response network (41).

### LS chromosomes contribute to the individual expansion of FOSC kinomes.

The conserved kinome represents defining pathways shared among *Ascomycetes* across evolutionary time. Our data support the hypothesis that the unique additions to each kinome enable species-specific adaptation. Among the species here, F. oxysporum kinomes are the largest. At roughly twice the size of the core ascomycete kinome, the average *Fusarium* kinome contains 185 kinases in 112 subfamilies (Table 1 and Table S2). In addition, we observed a positive correlation between kinases encoded in the LS region of each strain (defined as lineage-specific [LS] kinases hereafter) with the total number of LS genes (*y* = 0.0049*x* + 6.11; *R*^2^ = 0.57) (Fig. S1a), suggesting that LS chromosomes contribute directly to the expanded kinomes (Table S4). This correlation exhibits a slope similar to that of the whole genome, but effectively without a minimum kinase requirement.

10.1128/mSphere.00231-18.1 FIG S1  The LS genome contributes to kinome expansion. (a) The number of genes in the LS genome was plotted against the number of kinases found with the LS genome of each *Fusarium oxysporum* (Fo) strain. (b) The LS genome of each FOSC strain was compared to the LS genome of the reference strain. The colored lines indicate the regions of conservation. Genomes were mapped to core genome contig 14 (roughly the first 1/5th of chromosome 1, a conserved chromosome) as a control. Large regions of color indicate potentially shared LS genome contigs. Names listed at the top of the ring correspond to the Fo strain for that particular colored ring (i.e., FoCotton is listed as Cotton). For brevity, the Fo identifier was left off the strain ID labels. Chr., chromosome. Download FIG S1, PDF file, 0.3 MB. Copyright © 2018 DeIulio et al. 2018 DeIulio et al. This content is distributed under the terms of the Creative Commons Attribution 4.0 International license.

In agreement with a comparative study among three *Fusarium* genomes (2), little conservation was observed among the LS genomes (Fig. S1b). The genome of each FOSC strain was partitioned into core and lineage-specific genes using an “eliminating core” method (see Materials and Methods for details). On average, the coverage of shared sequences among core genomes is more than 70% (>90% sequence similarity), as illustrated by supercontig 14 from the core. The average overlap for LS chromosomes of reference strain 4287 was 6.3%. The most significant conservation was observed in chromosome 14 between two tomato pathogens, MN25 (race 3) and the reference genome Fo4287 (F. oxysporum 4287) (race 2) (Table S3). This closeness was the result of the transfer of a pathogenicity chromosome, chromosome 14, suggested by comparative studies (2, 42). Other conserved fragments were also observed; however, their functional significance remains elusive.

10.1128/mSphere.00231-18.8 TABLE S3  Lineage-specific genome contig overlap among FOSC strains. All LS supercontigs from Fo4287 were compared to all other species LS contigs using BLAST (see Materials and Methods for details). The percentage of each Fo4287 supercontig identified within each other FOSC strain is listed as a percentage of the total Fo4287 supercontig size. Supercontig 2.14, from the core genome, is included as a control. The Fo4287 chromosome from which the supercontig originated is listed at the left. Download TABLE S3, XLSX file, 0.01 MB. Copyright © 2018 DeIulio et al. 2018 DeIulio et al. This content is distributed under the terms of the Creative Commons Attribution 4.0 International license.

10.1128/mSphere.00231-18.9 TABLE S4  FOSC lineage-specific kinases. Kinases within genomic regions predicted to be of lineage-specific origin are listed by FOSC strain. Gene IDs are listed along with the supercontig number from that particular strain from which the kinase originated and the kinase family. A number next to the strain name at the left indicates the total number of LS kinases in that strain. Download TABLE S4, XLSX file, 0.02 MB. Copyright © 2018 DeIulio et al. 2018 DeIulio et al. This content is distributed under the terms of the Creative Commons Attribution 4.0 International license.

To evaluate the overall functional importance of LS kinases, we have sequenced mRNA isolated from the reference strain Fo4287 under two experimental conditions, one at room temperature and the other shifted to 37°C (see Materials and Methods for details) (Table S5). Roughly half of the annotated Fo4287 genes (9,914) were expressed in either condition, including 85% of kinases within the conserved ascomycete kinome (85 out of the total 99 core kinases). In contrast, among the 44 LS kinases of strain Fo4287, the expression of only 8 (18%) was detected. Expressed LS kinases include two HisK kinases (FOXG_14953 and FOXG_15045), two Atypical/FunK1 kinases (FOXG_12507 and FOXG_14032), two Other/HAL kinases (FOXG_06573 and FOXG_07253), one STE/YSK kinase (FOXG_14024), and one unclassified kinase (FOXG_16175). In most cases, we saw higher levels of expression for the copies in the core than for the LS copies.

10.1128/mSphere.00231-18.10 TABLE S5  Gene expression and differential expression across temperature shift in strain Fo4287. Gene expression information is listed for all annotated genes within the Fo4287 genome at the time of the experiment. Gene IDs are listed at the left with the first five columns to the right representing the FPKM value for that gene in each data set. Two replicates for 28°C and three replicates for 37°C are included. The seven columns adjacent to FPKM expression show the information provided by differential expression analysis under conditions for that gene. If a gene was not found to be differentially expressed, the adjusted *P* value was >0.05, “NOT_DIFFERENTIALLY_EXPRESSED” is listed, instead of software output information. Download TABLE S5, XLSX file, 1.6 MB. Copyright © 2018 DeIulio et al. 2018 DeIulio et al. This content is distributed under the terms of the Creative Commons Attribution 4.0 International license.

Interestingly, heat stress at 37°C uniquely induced the expression of 4,394 genes, accounting for 44% of all expressed genes (Table S5). Similarly, about 44% of all expressed core kinases are induced under heat stress, consistent with the functional conservation of the core genome. In contrast, six out of eight expressed LS kinases were induced at 37°C, including a HAL kinase (FOXG_06573), a class IV HisK (FOXG_14953), one Atypical/FunK1 (FOXG_12507), and three unclassified kinases. The overall expression pattern of the Fo4287 kinome supports the potential function of some LS kinases, especially in coping with different stresses. Additional functional studies under diverse stress conditions may capture the expression of other, potentially condition-specific, LS kinases.

### Expanded FOSC kinases enhance signaling transduction in cell cycle control and environmental sensing.

Expanded families belong to the category ii families (Fig. 1d), enhancing functions related to cell cycle control and environmental sensing. For a soilborne pathogen with strong host specificity, like F. oxysporum, the adjustment of growth and cell cycle control in response to environmental cues is likely essential for survival.

### (i) Enhanced cell cycle control centering on the TOR kinase.

**FIG 2:**
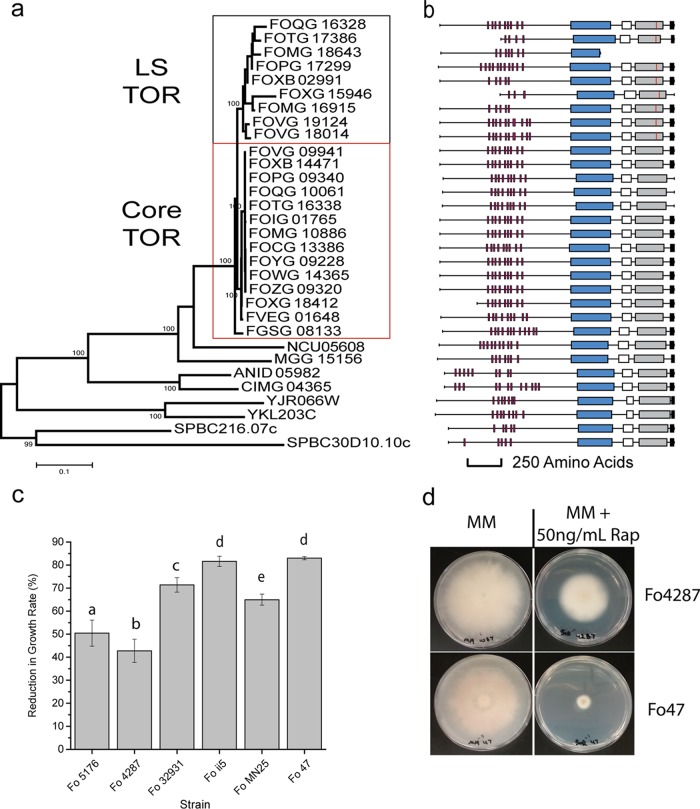
TOR kinase expansion through the LS genome. (a) A phylogenetic tree of TOR kinases constructed by the neighbor-joining (NJ) algorithm using TOR kinase protein sequence alignment based on the kinase domain. The core and LS clades of TOR kinase in *Fusarium oxysporum* are shown in boxes. (b) To the right of the tree is a protein domain image showing the relative structure of each TOR kinase. A red vertical line inside the kinase domain indicates the M2345L mutation. The domains shown are as follows: HEAT repeats (red), FAT domain (blue), RFB domain (white), kinase domain (gray), and FATC domain (black). (c) Measurement in reduction of radial growth of six F. oxysporum (Fo) strains to 50 ng/ml rapamycin on plates containing minimal medium (MM) amended with antibiotic compared to no-antibiotic MM plate controls. Three biological replicates were done for each treatment. Groups that are statistically different from one another using a *t* test comparison between all groups are indicated by the letters a to d. (d) Pictures of fungal growth on minimal medium with and without antibiotic (rapamycin [Rap]) at 7 days postinoculation. Plates for the most resistant (Fo4287) and least resistant (Fo47) strains are shown.

One of the most interesting expansions is the TOR kinase, which occurred in 7 out of 10 sequenced plant-pathogenic FOSC strains (Fig. 2). The TOR kinase is a top regulator of nutrient sensing that dictates cellular responses according to the levels of nutrients and oxygen (43). A member of the Atypical/FRAP (FKBP12-rapamycin-associated protein) kinase subfamily, the TOR kinase is highly conserved in nearly all eukaryotic organisms from fungi to humans with few exceptions (44). Even though there are two TOR paralogs in the S. cerevisiae and S. pombe genomes, almost all euascomycete fungal genomes have only one copy (45). Our study revealed TOR kinase expansion in 7 out of 12 sequenced FOSC strains, with 5 strains containing one copy and two strains containing two additional copies in addition to the single orthologous TOR kinase (Fig. 2a). The orthologous copies of the TOR kinase (indicated as Core TOR in Fig. 2a) form a monophyletic group with almost identical amino acid sequences (>99.9%). A total of nine TOR paralogs (indicated as LS TOR in Fig. 2a) clustered together with an average 95.9% amino acid identity compared to the orthologous copies and an average 97% amino acid identity among LS paralogs (Fig. S2), arguing against a recent duplication within an individual genome as a mechanism for the expansion of this gene family. However, there does appear to have been a recent duplication of one pair of the paralogs in a pea-pathogenic strain (FOVG_18014 and FOVG_19124).

10.1128/mSphere.00231-18.2 FIG S2  Nucleotide conservation among TOR kinases confirms single origin for LS TOR kinases. (a) The domain structure of the TOR kinase is shown with the M2345L marked as a red band. The percent amino acid conservation in 10-bp windows across a gap of deleted protein sequences for *Fusarium graminearum* (Fg), *Fusarium verticillioides* (Fv), and Fo4287 (purple), all FOSC core genome TOR kinases (blue), and FOSC LS genome TOR kinases (black) are shown above the graph. (b) The nucleotide identity between TOR kinases in fusaria was compared. Gene IDs are organized into LS, Core, and Fg and Fv at the bottom. Colors are relative representations of the nucleotide identity. Download FIG S2, PDF file, 0.5 MB. Copyright © 2018 DeIulio et al. 2018 DeIulio et al. This content is distributed under the terms of the Creative Commons Attribution 4.0 International license.

Six out of the nine TOR paralogs encode full-length proteins containing all functional domains (Fig. 2b). One of the paralogous copies in the melon pathogen lacked the kinase domain, and two paralogous copies (FOXG_15946 and FOTG_17386) had a truncation at the N terminus (part of the HEAT repeats) of the protein. With the highly conserved domain structure, we were able to identify a shared mutation, M2345 to lysine, among all TOR paralogs near the very end of the catalytic loop of the kinase domain, while all other sequence motifs essential for TOR function were conserved (Fig. 2b). On the basis of the protein structure, this catalytic loop forms the back of the ATP binding pocket, an invariant site for all conserved TOR kinases (46).

López-Berges et al. (47) reported that the truncation of FOXG_15946 was the result of a transposon insertion, and a very low level of expression of this truncated copy was detected in rich medium. Our transcriptome sequencing (RNA-seq) data on the *Arabidopsis* pathogen Fo5176 during the course of infection also support the expression of both the ortholog (FOXB_14471) and the expanded copy (FOXB_02991) of the TOR kinases, and the expression of the orthologous copy is roughly three times higher than that of the paralogous copy (L.-J. Ma, unpublished data).

As the TOR kinase is the direct target of the antibiotic rapamycin, we tested the sensitivity of six F. oxysporum strains, four strains with one TOR copy and two strains with two TOR copies, to rapamycin. Rapamycin (50 ng/ml) reduced the rate of growth of all strains (*P* < 0.005) (Fig. 2c and d; Fig. S3). For the four strains containing the single copy of the TOR kinase, reduction in growth rate ranged from between 64% (MN25) to 82% (Fo47). The two strains carrying an additional TOR kinase, strains Fo5176 and Fo4287, showed increased resistance to the rapamycin treatment, resulting in 50% and 42% reduced growth, respectively. These results are consistent with an additional TOR kinase providing enhanced resistance to the drug rapamycin.

10.1128/mSphere.00231-18.3 FIG S3  Differential inhibition of FOSC isolate growth by rapamycin. (Top) Images for one representative pair of plates were taken for each strain at 7 days postinoculation (dpi). Strains were cultured on minimal medium (MM) or minimal medium with 50 ng/ml rapamycin (MM +Rap). (Bottom) The growth rate (in millimeters per 24 h) was calculated for each strain on both media. All MM and MM plus rapamycin (RAP) pair differences are statistically significant (*P* value of <0.005 by *t* test). All plates were done in triplicate. Download FIG S3, PDF file, 0.2 MB. Copyright © 2018 DeIulio et al. 2018 DeIulio et al. This content is distributed under the terms of the Creative Commons Attribution 4.0 International license.

Functional studies suggested that the TOR signaling pathway may control pathogenic phenotypes, such as virulence in F. oxysporum infecting tomato (47) and toxin production in Fusarium fujikuori (45). Even though we do not have direct evidence on the specific function of the expansion of TOR kinase among FOSC, the high frequency (70% plant-pathogenic strains), the preserved protein domain organization, and the increased resistance to rapamycin all suggest its potential functional involvement in adaptation to diverse environments.

### (ii) Expansion of other signaling components involved in cell cycle control.

TOR and its complex modulate cell growth patterns by partnering with other signaling components to control a number of regulatory subnetworks (48). Interestingly, we observed the expansion of several kinase families functioning as TOR partners, including CDC2, CDC2-like kinase (CLK), and the BCK1 MAPK.

### *(a) BCK1*.

The *Fusarium* BCK1 kinase family is expanded among 8 out of the 10 plant-pathogenic isolates. All BCK1 kinases in FOSC fall into two clades, including an orthologous clade conserved in all *Fusarium* genomes, and an LS group found only in nine FOSC isolates (Fig. S4a). BCK1 is a member of the STE group kinase and is the only expanded family belonging to highly conserved category I families (Fig. 1d). Part of the MAPK signaling cascades under the control of the Rho1 GTPase and PKC1, BCK1 interacts with the TOR complex through direct binding with one of the subunits, LST8 (18, 20), and regulates transcription during the G_1_-to-S-phase transition as reported in S. cerevisiae (49).

10.1128/mSphere.00231-18.4 FIG S4  BCK1, CLK, CDC2, and HAL kinase subfamilies. Phylogenetic trees (maximum likelihood) using nucleotide sequences from BCK1 (a), CLK (b), CDC2 (c), and HAL (d) kinase subfamilies from all species show the clear separation of core and LS genome orthologs. The scale bar shows the substitution rate. LS genome genes (black boxes), core genome genes (white boxes), and unknown contigs (question marks) are indicated. Download FIG S4, PDF file, 0.9 MB. Copyright © 2018 DeIulio et al. 2018 DeIulio et al. This content is distributed under the terms of the Creative Commons Attribution 4.0 International license.

### *(b) CMGC families*.

Several kinase families of the CMGC group (Fig. 1b), including CDC2, CLK, and SRPKL kinases, are expanded in FOSC genomes. Similar to TOR and BCK1 kinases, CLK gene expansion was observed only among FOSC genomes. An LS CLK was present in 7 out of 10 sequenced FOSC plant-pathogenic strains. The core and LS CLKs are phylogenetically distinct with strong bootstrap support (Fig. S4b). However, the expansion of CDC2 (Fig. S4c) and SRPKL kinases (Table S2) had already occurred within the genus *Fusarium* before the split of *Fusarium* species. Collectively, SRPKL kinases constitute 4% to 7% of the total kinome among FOSC genomes (Table S2), and expansion happened multiple times. All other fungal genomes examined here contain a single CDC2 kinase, while F. graminearum, F. verticillioides, and all FOSC genomes have two. Expansion continued further within the FOSC. The tomato pathogen Fo4287 contains 10 additional LS CDC2 kinases, and the pea pathogen *F*. *oxysporum* HDV247 (FoHDV247) contains one extra copy. Not surprisingly, the two CDC2 genes detected in all *Fusarium* species are located in the core of the genome, and recent duplication events resulted in the 10 LS copies in the Fo4287 strain (2).

These expanded CMGC kinase families all have functions related to cell cycle control directly or indirectly linked through the TOR signaling pathways. The CDC2 kinase is a major regulator of the cell cycle. In S. pombe, the CDC2 kinase (SPBC11B10.09) directly regulates the G_1_ to S and G_2_ to M transitions and DNA damage repair (50, 51). CLK kinases are involved in various cellular functions, including regulating the cell cycle in S. pombe (52), controlling ribosome and tRNA synthesis in response to nutrient limitation and other cellular stresses in S. cerevisiae (53), and regulating cell wall biogenesis, vegetative growth, and sexual and asexual development in Aspergillus nidulans (54, 55). Like the CLK kinases, the SRPK kinases have been implicated in the control of SR protein-mediated splicing in a TOR-dependent manner in S. cerevisiae (56).

Functional importance of these expanded kinases in *Fusarium* genomes has been indicated by a few functional studies; although only a few functional studies have been performed, they were solid studies. Deletion of the orthologous CDC2 kinases (FGSG_08468) in F. graminearum resulted in profound pleiotropic effects, including reduced virulence and decreased ascospore production. Deletion of the *Fusarium*-specific copy (FGSG_03132) resulted in a milder, but still significant phenotype (15). In F. graminearum, the CLK kinase was downregulated during conidial germination (15). Removal of one SRPKL kinase in F. graminearum (FGSG_02488 and SRPKL2) reduced DON production by half (15). The expression of the same gene was suppressed significantly during sexual development. Among the nine core SRPKL kinases, five were expressed and four (two SRPKL1 kinases [FOXG_08977 and FOXG_10022], one SRPKL1 kinase [FOXG_21922], and one SRPKL3 kinase [FOXG_19803]) were upregulated at 37°C. Two CDC2 kinases within the core were expressed under both conditions, while expression of the 10 LS copies was not detected. Of the 51 unclassified kinases in strain Fo4287, expression of 12 core kinases and one LS kinase (FOXG_16175) was detected in the RNA-seq data generated from the reference strain.

### (iii) Enhanced environmental sensing accomplished through HisKs.

**FIG 3:**
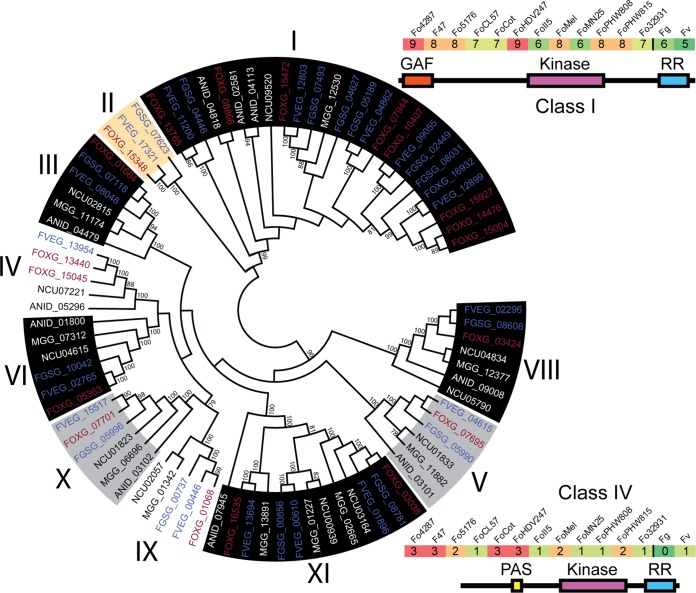
Histidine kinases are expanded in the fusaria. A phylogenetic tree was constructed for all two-component signaling histidine kinase nucleotide sequences and labeled according to their class (family) (neighbor-joining algorithm). Some kinases were excluded, as they lacked a large portion of the aligned conserved sequence among HisKs. Kinases that are part of the conserved ascomycete kinome (gray background), filamentous kinome (black background), and *Fusarium* kinome (white background) and kinases that are found only in fusaria in this study (orange background) are indicated. *Fusarium oxysporum* (Fo) gene identifiers (IDs) are colored red, and *Fusarium graminearum* (Fg) and *Fusarium verticillioides* (Fv) gene IDs are colored blue. All bootstrap values above 80% are shown. The domain structure for the class I and class IV HisKs are shown along with a heatmap of the copy number for each class among the fusaria.

One of the most significant kinase expansions occurs in the HisK group (Fig. 3) known to play important roles in sensing and transducing many intra- and extracellular signals (36–39). The two yeast genomes (S. cerevisiae and S. pombe) contain gene(s) encoding one and three HisKs, respectively, while the HisK group consistently expands across the filamentous fungi (Fig. 1d). All filamentous ascomycete fungal genomes included in this study have more than 10 HisKs, and FOSC isolates have by far the most HisKs, with the tomato wilt pathogen Fol4287 and the tomato root rot pathogen FoHDV247 both predicted to have 23 HisKs. Based on a classical classification, fungal HisKs are divided into 11 classes (36). Excluding F. graminearum which lacks the class IV HisK (TcsA kinase), all *Fusarium* genomes contain 10 distinct classes of HisK, only lacking the class VII that was reported in the Cochliobolus heterostrophus and Botrytis cinerea genomes (36). Interestingly, the class II HisK is uniquely present in all *Fusarium* genomes and few other plant-pathogenic *Ascomycetes*, including C. heterostrophus and Bipolaris maydis (36). The most significantly expanded FOSC HisKs are in class I and class IV (Fig. 3), for instance, class I HisKs continue to expand from 5 in F. verticillioides and 6 in F. graminearum to 7 or more among FOSC genomes.

According to protein domain organization, class I and class IV contain a GAF domain and a PAS (Per-Arnt-Sim) N-terminal domain, respectively. PAS domains detect signals, including light and oxygen, usually through binding to an associated cofactor (57). GAF domains form small binding pockets with potentials to bind to small signal molecules, such as cyclic GMP, cyclic AMP, or both. The motif is ubiquitously present in hundreds of signaling and sensory proteins from all kingdoms of life (58). Between class I HisKs, amino acid conservation of the GAF domain is low, likely indicating nonredundant function. Unlike class I HisK, class IV copies have very similar N-terminal domain sequences. Other than class I HisKs, class II, VIII, X, and XI HisKs also contain GAF-related domains, while class IV, V, IX, and XI HisKs also contain PAS-related domains.

First reported in prokaryotes, bacterial HisKs involve two components where the first component senses environmental signals, autophosphorylates a conserved histidine residue, and then transduces the signal to the second component through phosphate transfer (59). Among almost all eukaryotic systems, these two components are fused to form hybrid HisKs, containing the conserved histidine residue, ATP binding and response regulator (RR) receiver domains functioning in three-step “phosphorelay” reactions. Upon activation, HisK phosphorylates itself twice, ending with phosphorylation of the response receiver domain at the C terminus. This phosphate is then transferred via a histidine phosphotransfer (Hpt) protein to a response regulator, which carries out downstream signaling functions. Each *Fusarium* isolate was found to have two RR proteins, orthologous to the yeast SKN7 and SSK1 RR proteins that are both involved in the osmotic stress response downstream from the SLN1 HisK and YPD1 Hpt proteins (60, 61). The single yeast Hpt protein YPD1 has a single counterpart in F. graminearum, F. verticillioides, and each FOSC strain except for strains FoPHW808, FoPHW815, FoMelon, and FoCL57 (see Table 1 for strain abbreviation explanations), which each have two. In these four cases, the strains contain one copy that is located within the core genome and one copy that is located within the LS genome. Core genome copies share a 100% amino acid identity across the 143-amino-acid (aa) protein, while LS genome copies share roughly 91% amino acid identity across 154 aa.

### (iv) Other expanded kinases. *(a) HAL kinases*.

Regulators of the cell's primary potassium pumps (62, 63), the HAL kinases, are expanded in 6 out of the 12 sequenced FOSC genomes. In addition to the orthologous copies located in core genome regions, the second phylogenetically supported group contains all LS HAL kinases. Different from other FOSC-specific expansions, this subfamily is also expanded in Magnaporthe oryzae and A. nidulans (Fig. S4d). Noticeably, the HAL kinases are most significantly expanded to six copies in strain Fo32931, a F. oxysporum strain isolated from an immunocompromised patient. Among the five HAL kinases encoded by genes in the reference genome Fo4287, all except one (FOXG_04685) were expressed (no change of expression with the shift of temperature), but expression of the single core genome HAL kinase was at least 30-fold higher than expression of the LS HAL kinases.

### *(b) Unclassified protein kinases*.

In addition, we observed a large number of kinases in the unclassified family among all *Fusarium* genomes. On average, each *Fusarium* genome contains 41 unclassified kinases, accounting for roughly half of the kinome beyond the core ascomycete kinome and constituting 30% to 50% of the LS kinome among FOSC genomes. No unclassified kinases were present among all ascomycete fungal genomes examined, but four are conserved within the genus *Fusarium* (not shown) and more conservation was observed among FOSC genomes (Fig. S5). Among these unclassified kinases, eight were included in a F. graminearum knockout study and six mutants exhibit measurable phenotypes (15), including decreased production of mycotoxin deoxynivalenol in five mutants (FGSG_13509, FGSG_02153, FGSG_00792, FGSG_06420, and FGSG_10591), and significant upregulation during sexual development for another (FGSG_12132).

10.1128/mSphere.00231-18.5 FIG S5  Shared unclassified kinases among the FOSC. Phylogenetic trees (maximum likelihood) using nucleotide sequences from all FOSC unclassified kinases. Bars around the outside of the tree denote clades supported with high bootstrap values. When two clades are adjacent, one is colored blue as a visual aid. The number next to each bar indicates the number of FOSC strains that contain a gene from that clade. Download FIG S5, PDF file, 0.5 MB. Copyright © 2018 DeIulio et al. 2018 DeIulio et al. This content is distributed under the terms of the Creative Commons Attribution 4.0 International license.

### *(c) Atypical/FunK1*.

Among the expanded families, one of the least understood is the Atypical/FunK1, found only in complex *Basidiomycota* and *Pezizomycota* and possibly involved in the switch to multicellularity (64). Among fusaria, copy number ranged from a single copy to nine copies, including one that is highly conserved among all *Fusarium* genomes. The expression of this conserved FunK1 gene in F. graminearum (FGSG_03499) is strongly upregulated during sexual development (15). Since no sexual reproduction was reported in F. oxysporum, the conservation of this gene within the *Fusarium* genus suggests functions other than sexual reproduction.

## DISCUSSION

Kinases play key roles in transmitting external and internal signals and regulating complex cellular signaling responses. Genomes for fungi in the genus *Fusarium* have large kinomes compared to those of most fungi. As it is crucial for a pathogen to adapt to stresses encountered both outside and inside its host, it is not surprising to see expansion of kinases among FOSC, as the species complex thrives in diverse hosts. The positive correlation between the total number of proteins and the size of the fungal kinome reported here was also reported in pathogenic *Microsporidia* species and several model organisms (10). Distinctively, this study defined a core kinome of the ascomycete fungi consisting of about 100 kinases, in agreement with a prediction based on gene to kinase count. This core kinome outlines the most fundamental kinase signaling pathways supporting the largest phylum of fungi.

Within the ascomycete fungal lineages, some kinase families are more recalcitrant to change. The MAP kinase cascades, cell kinase I, and calcium/calmodulin-regulated kinases changed very little within the phylum over millions of years of evolutionary time. However, we observed the expansion of cyclin-dependent kinases and other close relatives, histidine kinases, and Atypical kinases, suggesting their potential roles in species-specific adaptation along the various evolutionary trajectories of ascomycete fungi.

The number of unclassified kinases increased significantly within the genus *Fusarium* and made up a majority of the kinases found within FOSC kinomes beyond those conserved among the filamentous fungi. In contrast, most non-*Fusarium* filamentous fungi had relatively few unclassified kinases. Additional research is necessary to understand how these unclassified kinases contribute to *Fusarium*-specific evolution and adaptation. However, it is clear that many unclassified kinases are important, judging by the results from a reverse genetic screen (15), observed levels of expression in our RNA-seq data, and their conservation within the genus and across the FOSC.

Both the histidine kinase groups and the SPRKL family kinases were largely absent from the yeasts, moderately expanded in the non-*Fusarium* filamentous fungi, and appeared in large numbers in the FOSC. Little is known about the function of SRPKL kinases in fungal biology. HisKs are extensively used by bacteria and archaea to sense and respond to a variety of biotic and abiotic stimuli (65). In addition to regulating stress responses as reported in yeasts, the filamentous class III HisKs were reported to regulate fungal morphogenesis and virulence in various human-, plant-, and insect-pathogenic fungi (for a detailed review, see reference 37). Overall, the function of histidine kinases is understudied in filamentous fungi. As HisKs are absent in mammals and some are essential for virulence in fungal pathogens, they represent interesting fungal targets for the discovery of new antifungal drugs as illustrated by Shor and Chauhan (66). Although limited functional studies exist for HisK or SRPKL kinases, the continuous expansion suggests the functional importance of these understudied protein kinases. A better understanding of their functions would not only inform *Fusarium* biology but also could be extrapolated to other filamentous fungi and complex basidiomycetes.

Although LS kinases differ from their core counterparts, catalytic domains are generally conserved. For BCK1 kinases, the catalytic kinase domain of all nine LS BCK1 kinases is highly conserved (~91% between groups), but all of them are roughly 800 aa shorter than their core genome counterparts, missing a portion of the N terminus. Similarly, the sequences of the additional copies of CLK differ significantly from those of the orthologous copy; however, the invariant residues within the catalytic kinase domain were conserved among all. In the case of CDC2 kinases, two of the three phosphorylation sites, Y15 and T14, are mutated in all LS CDC2 kinases. Since phosphorylation of these residues inhibits CDC2 kinase function (67), mutation at these two residues may lead to CDC2 kinase activity without tight control.

Most interestingly, we observed the repeated expansion of TOR kinase among the FOSC genomes. Most fungi have a single TOR protein; however, two TOR paralogs were observed in the genomes of both S. cerevisiae and S. pombe yeast (43). Duplication of TOR kinases was also reported in Batrachochytrium dendrobatidis, an amphibian-pathogenic chytrid (44). Seated at the center of many signal transduction pathways, TOR integrates the input from upstream pathways, sensing cellular nutrient, oxygen, and energy levels, and dictates cellular responses. The convergent evolution toward TOR kinase duplication in the fungal kingdom might reflect selection for more finely tuned environmental response pathways. Interestingly, we found that two FOSC strains containing an additional TOR copy were more resistant to rapamycin that those containing only one copy. Although our data for strain Fo4287 indicated that the TOR paralog was unexpressed, it may be expressed under other, unobserved, conditions.

The expansion of families like the TOR kinase has been, in many cases, facilitated in part by the LS genome, that contributes to the unique and specific expansion of subfamilies, such as HisK, CLK, Atypical/FunK1, HAL, CMGC/SRPKL, and Atypical/FRAP. Many of these kinases have either a known or proposed function in responding to environmental signals or cell cycle control and in many cases function downstream of TOR. Most expanded subfamilies can be linked to environmental signaling or cell cycle control; many of these kinases are under control of the TOR nutrient-sensing complexes. Through the lenses of kinases, these pathogens seem to be enhancing their ability to sense their environment and tighten their regulation of the cell cycle, mediated primarily through the TOR signaling pathways.

## MATERIALS AND METHODS

### Generation of fungal kinomes.

The two yeast genomes Saccharomyces cerevisiae S288C (Sc), Schizosaccharomyces pombe strain 972h- (Spom) were downloaded from the National Center for Biotechnology Information (NCBI). All other fungal genomes were downloaded from The Broad Institute of MIT and Harvard and used for kinome analysis. Using the established Kinannote pipeline, we generated the kinomes of the above fungal genomes. Briefly, Kinannote uses hidden Markov models generated from the manually aligned complete kinome of the slime mold Dictyostelium discoideum to search the given genome for kinases. It then identifies both well-conserved eukaryotic protein kinases as well as unusual protein kinases. Finally, Kinannote uses BLAST search results to classify the kinases based on family.

Kinannote assigned all kinases in all genomes into 135 different classes (see Table S1 in the supplemental material). To create the conserved kinomes, we removed any kinase subfamilies for which, under a given phylogenetic grouping, more than a single species was missing the family entirely. The groupings consisted of all species (*Ascomycetes*), all species except S. cerevisiae and S. pombe (filamentous fungi), only the genus *Fusarium*, and only members of the FOSC. The number of conserved subfamily members was set at the lowest number among all species, excluding one.

### Gene alignment and BLAST.

Fungal kinase protein and nucleotide sequences were downloaded from either the Broad Institute or NCBI. Sequences were aligned using either Muscle or Clustal through MEGA6 (68). Alignments were inspected manually and adjusted based on known conserved sequences. Sequence phylogeny was constructed using maximum likelihood and bootstrapped using 100 replicates.

BLAST was used to search for orthologs among the *Fusarium oxysporum* species complex (FOSC) strains, conserved lineage-specific (LS) kinases, and to identify conserved regions of the LS genome. Homologs were considered top BLAST hits that had more than 90% nucleotide identity and covered the entire genomic gene sequence. To find groups of conserved LS kinases, a file containing all LS kinase sequences from all strains was compared to an identical file using BLAST. A custom Perl script was generated to find and parse through results to identify groups of kinases that had significant BLAST results to each other. Identification of conserved LS regions was done by comparing each FOSC genome to the full LS genome of strain Fo4287, including one core supercontig as a control for mapping (supercontig 14). BLAST results were filtered for regions matching greater than 90% nucleotide identity and alignment lengths of greater than 3 kb. BLAST results were mapped to the Fo4287 LS reference sequence using BRIGS (69). Total overlap of each strain's LS region with that of strain Fo4287 was calculated by summing all regions returned by BLAST.

### Generation of RNA samples and data analysis.

Fusarium oxysporum f. sp. lycopersici 4287 (Fo4287) gene expression during temperature stress was assayed using transcriptome sequencing (RNA-seq). Fusarium oxysporum spores (1 × 10^9^ spores) were cultured for 14 h at 28°C (in the case of the 28°C growth experiment) or for 10 h at 28°C and switched to 37°C for 4 h (in the case of the 37°C growth experiment) in 200 ml of minimal medium supplemented with 25 mM sodium glutamate and buffered with HEPES (final concentration of 20 mM) to pH 7.4 at 170 rpm. Three replicates of each condition were done for each species, with 36 RNA samples in total. Fungal tissue was collected with filter paper, and RNA was extracted using a standard TRIzol RNA isolation reagent extraction (Life Technologies, Carlsbad, CA). The RNA library was constructed using Illumina TruSeq Stranded mRNA Library Prep kit (Illumina, CA) following the manufacturer's protocol and sequenced using the Illumina HiSeq platform (Illumina, CA).

Resulting data files were trimmed using Trimmomatic to remove poor-quality reads (70). Reads were then aligned into BAM (binary version of SAM) files using Rsubread (71). Gene expression and differentially expressed gene calls were then made using limma and edgeR (72, 73). Genes were called as expressed if both replicates had a reads per kilobase per million (RPKM) value greater than 1.0. Genes were called as differentially expressed genes (DEGs) if the adjusted Pearson correlation value was less than 0.05.

### Rapamycin resistance screen.

Cultures of strains Fo4287, Fo5276, Fo47, Foii5, and Fo32931 were grown in potato dextrose broth (Becton, Dickinson and Company, Sparks, MD), strain MN25 was grown on potato dextrose agar (Becton, Dickinson and Company, Sparks, MD) for 5 days, The strains were spotted into the center of plates containing either minimal medium alone (74) or minimal medium supplemented with rapamycin (final concentration of 50 ng/ml). The plates were stored at 28° for 48 h and then transferred to room temperature. After the transfer to room temperature, the diameter of each colony was measured at 24-h intervals. Growth rate was determined as the average increase in size (in millimeters) per 24 h. Reduction in growth rate was calculated as follows: 100 − [(growth rate on rapamycin-containing plate/growth rate on control plate) × 100]. In order to generate a range of error, all nine comparisons of growth rate between the three control plates and three rapamycin-containing plates were used to calculate reduction in growth. All species/condition plates were done in triplicate.

### Data availability.

The Fusarium oxysporum temperature RNA-seq data for this study have been deposited into the NCBI GEO repository under accession number GSE113332.

## References

[1] Ma L-J, Geiser DM, Proctor RH, Rooney AP, O'Donnell K, Trail F, Gardiner DM, Manners JM, Kazan K 2013 Fusarium pathogenomics. Annu Rev Microbiol 67:399–416. doi:10.1146/annurev-micro-092412-155650. 24024636

[2] Ma LJ, van der Does HC, Borkovich KA, Coleman JJ, Daboussi MJ, Di Pietro A, Dufresne M, Freitag M, Grabherr M, Henrissat B, Houterman PM, Kang S, Shim WB, Woloshuk C, Xie X, Xu JR, Antoniw J, Baker SE, Bluhm BH, Breakspear A, Brown DW, Butchko RA, Chapman S, Coulson R, Coutinho PM, Danchin EG, Diener A, Gale LR, Gardiner DM, Goff S, Hammond-Kosack KE, Hilburn K, Hua-Van A, Jonkers W, Kazan K, Kodira CD, Koehrsen M, Kumar L, Lee YH, Li L, Manners JM, Miranda-Saavedra D, Mukherjee M, Park G, Park J, Park SY, Proctor RH, Regev A, Ruiz-Roldan MC, Sain D, Sakthikumar S, et al. 2010 Comparative genomics reveals mobile pathogenicity chromosomes in Fusarium. Nature 464:367–373. doi:10.1038/nature08850. 20237561 PMC3048781

[3] Vlaardingerbroek I, Beerens B, Schmidt SM, Cornelissen BJC, Rep M 2016 Dispensable chromosomes in Fusarium oxysporum f. sp. lycopersici. Mol Plant Pathol 17:1455–1466. doi:10.1111/mpp.12440. 27271322 PMC6638487

[4] Schmidt SM, Houterman PM, Schreiver I, Ma L, Amyotte S, Chellappan B, Boeren S, Takken FLW, Rep M 2013 MITEs in the promoters of effector genes allow prediction of novel virulence genes in Fusarium oxysporum. BMC Genomics 14:119. doi:10.1186/1471-2164-14-119. 23432788 PMC3599309

[5] Smith DA, Morgan BA, Quinn J 2010 Stress signalling to fungal stress-activated protein kinase pathways. FEMS Microbiol Lett 306:1–8. doi:10.1111/j.1574-6968.2010.01937.x. 20345377 PMC3644883

[6] Martin H, Shales M, Fernandez-Piñar P, Wei P, Molina M, Fiedler D, Shokat KM, Beltrao P, Lim W, Krogan NJ 2015 Differential genetic interactions of yeast stress response MAPK pathways. Mol Syst Biol 11:800. doi:10.15252/msb.20145606. 25888283 PMC4422557

[7] Turrà D, Segorbe D, Di Pietro A 2014 Protein kinases in plant-pathogenic fungi: conserved regulators of infection. Annu Rev Phytopathol 52:267–288. doi:10.1146/annurev-phyto-102313-050143. 25090477

[8] Kosti I, Mandel-Gutfreund Y, Glaser F, Horwitz BA 2010 Comparative analysis of fungal protein kinases and associated domains. BMC Genomics 11:133. doi:10.1186/1471-2164-11-133. 20178650 PMC2838846

[9] Hindle MM, Martin SF, Noordally ZB, van Ooijen G, Barrios-Llerena ME, Simpson TI, Le Bihan T, Millar AJ 2014 The reduced kinome of Ostreococcus tauri: core eukaryotic signalling components in a tractable model species. BMC Genomics 15:1–21. doi:10.1186/1471-2164-15-640. 25085202 PMC4143559

[10] Li Z, Hao Y, Wang L, Xiang H, Zhou Z 2014 Genome-wide identification and comprehensive analyses of the kinomes in four pathogenic Microsporidia species. PLoS One 9:e115890. doi:10.1371/journal.pone.0115890. 25549259 PMC4280135

[11] Goldberg JM, Manning G, Liu A, Fey P, Pilcher KE, Xu Y, Smith JL 2006 The Dictyostelium kinome—analysis of the protein kinases from a simple model organism. PLoS Genet 2:e38. doi:10.1371/journal.pgen.0020038. 16596165 PMC1420674

[12] Miranda-Saavedra D, Stark MJ, Packer JC, Vivares CP, Doerig C, Barton GJ 2007 The complement of protein kinases of the microsporidium Encephalitozoon cuniculi in relation to those of Saccharomyces cerevisiae and Schizosaccharomyces pombe. BMC Genomics 8:309. doi:10.1186/1471-2164-8-309. 17784954 PMC2078597

[13] Manning G, Whyte DB, Martinez R, Hunter T, Sudarsanam S 2002 The protein kinase complement of the human genome. Science 298:1912–1934. doi:10.1126/science.1075762. 12471243

[14] Manning G, Plowman GD, Hunter T, Sudarsanam S 2002 Evolution of protein kinase signaling from yeast to man. Trends Biochem Sci 27:514–520. doi:10.1016/S0968-0004(02)02179-5. 12368087

[15] Wang C, Zhang S, Hou R, Zhao Z, Zheng Q, Xu Q, Zheng D, Wang G, Liu H, Gao X, Ma J-W, Kistler HC, Kang Z, Xu J-R 2011 Functional analysis of the kinome of the wheat scab fungus Fusarium graminearum. PLoS Pathog 7:e1002460. doi:10.1371/journal.ppat.1002460. 22216007 PMC3245316

[16] De Souza CP, Hashmi SB, Osmani AH, Andrews P, Ringelberg CS, Dunlap JC, Osmani SA 2013 Functional analysis of the Aspergillus nidulans kinome. PLoS One 8:e58008. doi:10.1371/journal.pone.0058008. 23505451 PMC3591445

[17] Coito C, Diamond DL, Neddermann P, Korth MJ, Katze MG 2004 High-throughput screening of the yeast kinome: identification of human serine/threonine protein kinases that phosphorylate the hepatitis C virus NS5A protein. J Virol 78:3502–3513. doi:10.1128/JVI.78.7.3502-3513.2004. 15016873 PMC371080

[18] Sharifpoor S, van Dyk D, Costanzo M, Baryshnikova A, Friesen H, Douglas AC, Youn J-Y, VanderSluis B, Myers CL, Papp B, Boone C, Andrews BJ 2012 Functional wiring of the yeast kinome revealed by global analysis of genetic network motifs. Genome Res 22:791–801. doi:10.1101/gr.129213.111. 22282571 PMC3317160

[19] Bharucha N, Ma J, Dobry CJ, Lawson SK, Yang Z, Kumar A 2008 Analysis of the yeast kinome reveals a network of regulated protein localization during filamentous growth. Mol Biol Cell 19:2708–2717. doi:10.1091/mbc.E07-11-1199. 18417610 PMC2441683

[20] Breitkreutz A, Choi H, Sharom JR, Boucher L, Neduva V, Larsen B, Lin Z-Y, Breitkreutz B-J, Stark C, Liu G, Ahn J, Dewar-Darch D, Reguly T, Tang X, Almeida R, Qin ZS, Pawson T, Gingras A-C, Nesvizhskii AI, Tyers M 2010 A global protein kinase and phosphatase interaction network in yeast. Science 328:1043–1046. doi:10.1126/science.1176495. 20489023 PMC3983991

[21] Mok J, Kim PM, Lam HYK, Piccirillo S, Zhou X, Jeschke GR, Sheridan DL, Parker SA, Desai V, Jwa M, Cameroni E, Niu H, Good M, Remenyi A, Ma J-LN, Sheu Y-J, Sassi HE, Sopko R, Chan CSM, De Virgilio C, Hollingsworth NM, Lim WA, Stern DF, Stillman B, Andrews BJ, Gerstein MB, Snyder M, Turk BE 2010 Deciphering protein kinase specificity through large-scale analysis of yeast phosphorylation site motifs. Sci Signal 3:ra12. doi:10.1126/scisignal.2000482. 20159853 PMC2846625

[22] Loewith R, Hall MN 2011 Target of rapamycin (TOR) in nutrient signaling and growth control. Genetics 189:1177–1201. doi:10.1534/genetics.111.133363. 22174183 PMC3241408

[23] Galagan JE, Calvo SE, Borkovich KA, Selker EU, Read ND, Jaffe D, FitzHugh W, Ma LJ, Smirnov S, Purcell S, Rehman B, Elkins T, Engels R, Wang S, Nielsen CB, Butler J, Endrizzi M, Qui D, Ianakiev P, Bell-Pedersen D, Nelson MA, Werner-Washburne M, Selitrennikoff CP, Kinsey JA, Braun EL, Zelter A, Schulte U, Kothe GO, Jedd G, Mewes W, Staben C, Marcotte E, Greenberg D, Roy A, Foley K, Naylor J, Stange-Thomann N, Barrett R, Gnerre S, Kamal M, Kamvysselis M, Mauceli E, Bielke C, Rudd S, Frishman D, Krystofova S, Rasmussen C, Metzenberg RL, Perkins DD, Kroken S, Cogoni C, et al. 2003 The genome sequence of the filamentous fungus Neurospora crassa. Nature 422:859–868. doi:10.1038/nature01554. 12712197

[24] Goldberg JM, Griggs AD, Smith JL, Haas BJ, Wortman JR, Zeng Q 2013 Kinannote, a computer program to identify and classify members of the eukaryotic protein kinase superfamily. Bioinformatics 29:2387–2394. doi:10.1093/bioinformatics/btt419. 23904509 PMC3777111

[25] Widmann C, Gibson S, Jarpe MB, Johnson GL 1999 Mitogen-activated protein kinase: conservation of a three-kinase module from yeast to human. Physiol Rev 79:143–180. doi:10.1152/physrev.1999.79.1.143. 9922370

[26] Robinson LC, Hubbard EJ, Graves PR, DePaoli-Roach AA, Roach PJ, Kung C, Haas DW, Hagedorn CH, Goebl M, Culbertson MR 1992 Yeast casein kinase I homologues: an essential gene pair. Proc Natl Acad Sci U S A 89:28–32. doi:10.1073/pnas.89.1.28. 1729698 PMC48168

[27] Tamuli R, Kumar R, Deka R 2011 Cellular roles of neuronal calcium sensor-1 and calcium/calmodulin-dependent kinases in fungi. J Basic Microbiol 51:120–128. doi:10.1002/jobm.201000184. 21077122

[28] Taylor SS, McKeon F 1997 Kinetochore localization of murine Bub1 is required for normal mitotic timing and checkpoint response to spindle damage. Cell 89:727–735. doi:10.1016/S0092-8674(00)80255-X. 9182760

[29] Lundgren K, Walworth N, Booher R, Dembski M, Kirschner M, Beach D 1991 mik1 and wee1 cooperate in the inhibitory tyrosine phosphorylation of cdc2. Cell 64:1111–1122. doi:10.1016/0092-8674(91)90266-2. 1706223

[30] Donaldson AD, Fangman WL, Brewer BJ 1998 Cdc7 is required throughout the yeast S phase to activate replication origins. Genes Dev 12:491–501. doi:10.1101/gad.12.4.491. 9472018 PMC316537

[31] Pearce LR, Komander D, Alessi DR 2010 The nuts and bolts of AGC protein kinases. Nat Rev Mol Cell Biol 11:9–22. doi:10.1038/nrm2822. 20027184

[32] Sobko A 2006 Systems biology of AGC kinases in fungi. Sci STKE 2006:re9. doi:10.1126/stke.3522006re9. 16971477

[33] Baldin C, Valiante V, Krüger T, Schafferer L, Haas H, Kniemeyer O, Brakhage AA 2015 Comparative proteomics of a tor inducible Aspergillus fumigatus mutant reveals involvement of the Tor kinase in iron regulation. Proteomics 15:2230–2243. doi:10.1002/pmic.201400584. 25728394

[34] Malumbres M 2014 Cyclin-dependent kinases. Genome Biol 15:122. doi:10.1186/gb4184. 25180339 PMC4097832

[35] Hamel L-P, Nicole M-C, Duplessis S, Ellis BE 2012 Mitogen-activated protein kinase signaling in plant-interacting fungi: distinct messages from conserved messengers. Plant Cell 24:1327–1351. doi:10.1105/tpc.112.096156. 22517321 PMC3398478

[36] Catlett NL, Yoder OC, Turgeon BG 2003 Whole-genome analysis of two-component signal transduction genes in fungal pathogens. Eukaryot Cell 2:1151–1161. doi:10.1128/EC.2.6.1151-1161.2003. 14665450 PMC326637

[37] Defosse TA, Sharma A, Mondal AK, Dugé de Bernonville T, Latgé JP, Calderone R, Giglioli-Guivarc'h N, Courdavault V, Clastre M, Papon N 2015 Hybrid histidine kinases in pathogenic fungi. Mol Microbiol 95:914–924. doi:10.1111/mmi.12911. 25560420

[38] Li D, Agrellos OA, Calderone R 2010 Histidine kinases keep fungi safe and vigorous. Curr Opin Microbiol 13:424–430. doi:10.1016/j.mib.2010.04.007. 20542727

[39] Santos JL, Shiozaki K 2001 Fungal histidine kinases. Sci STKE 2001:re1. doi:10.1126/stke.2001.98.re1. 11752677

[40] Buck V, Quinn J, Soto Pino T, Martin H, Saldanha J, Makino K, Morgan BA, Millar JB 2001 Peroxide sensors for the fission yeast stress-activated mitogen-activated protein kinase pathway. Mol Biol Cell 12:407–419. doi:10.1091/mbc.12.2.407. 11179424 PMC30952

[41] Pancaldi V, Saraç OS, Rallis C, McLean JR, Převorovský M, Gould K, Beyer A, Bähler J 2012 Predicting the fission yeast protein interaction network. G3 2:453–467. doi:10.1534/g3.111.001560. 22540037 PMC3337474

[42] van Dam P, Fokkens L, Schmidt SM, Linmans JHJ, Kistler HC, Ma L-J, Rep M 2016 Effector profiles distinguish formae speciales of Fusarium oxysporum. Environ Microbiol 18:4087–4102. doi:10.1111/1462-2920.13445. 27387256

[43] Heitman J, Movva NR, Hall MN 1991 Targets for cell cycle arrest by the immunosuppressant rapamycin in yeast. Science 253:905–909. doi:10.1126/science.1715094. 1715094

[44] Shertz CA, Bastidas RJ, Li W, Heitman J, Cardenas ME 2010 Conservation, duplication, and loss of the Tor signaling pathway in the fungal kingdom. BMC Genomics 11:510. doi:10.1186/1471-2164-11-510. 20863387 PMC2997006

[45] Teichert S, Wottawa M, Schönig B, Tudzynski B 2006 Role of the Fusarium fujikuroi TOR kinase in nitrogen regulation and secondary metabolism. Eukaryot Cell 5:1807–1819. doi:10.1128/EC.00039-06. 17031002 PMC1595341

[46] Sauer E, Imseng S, Maier T, Hall MN 2013 Conserved sequence motifs and the structure of the mTOR kinase domain. Biochem Soc Trans 41:889–895. doi:10.1042/BST20130113. 23863151

[47] López-Berges MS, Rispail N, Prados-Rosales RC, Di Pietro A 2010 A nitrogen response pathway regulates virulence functions in Fusarium oxysporum via the protein kinase TOR and the bZIP protein MeaB. Plant Cell 22:2459–2475. doi:10.1105/tpc.110.075937. 20639450 PMC2929112

[48] Laplante M, Sabatini DM 2009 mTOR signaling at a glance. J Cell Sci 122:3589–3594. doi:10.1242/jcs.051011. 19812304 PMC2758797

[49] Madden K, Sheu YJ, Baetz K, Andrews B, Snyder M 1997 SBF cell cycle regulator as a target of the yeast PKC-MAP kinase pathway. Science 275:1781–1784. doi:10.1126/science.275.5307.1781. 9065400

[50] Den Haese GJ, Walworth N, Carr AM, Gould KL 1995 The Wee1 protein kinase regulates T14 phosphorylation of fission yeast Cdc2. Mol Biol Cell 6:371–385. doi:10.1091/mbc.6.4.371. 7626804 PMC301198

[51] Caspari T, Hilditch V 2015 Two distinct Cdc2 pools regulate cell cycle progression and the DNA damage response in the fission yeast S. pombe. PLoS One 10:e0130748. doi:10.1371/journal.pone.0130748. 26131711 PMC4488491

[52] Yu EY, Lee JH, Kang WH, Park YH, Kim L, Park HM 2013 Fission yeast LAMMER kinase Lkh1 regulates the cell cycle by phosphorylating the CDK-inhibitor Rum1. Biochem Biophys Res Commun 432:80–85. doi:10.1016/j.bbrc.2013.01.082. 23376070

[53] Lee J, Moir RD, McIntosh KB, Willis IM 2012 TOR signaling regulates ribosome and tRNA synthesis via LAMMER/Clk and GSK-3 family kinases. Mol Cell 45:836–843. doi:10.1016/j.molcel.2012.01.018. 22364741 PMC3319249

[54] Kang E-H, Kim J-A, Oh H-W, Park H-M 2013 LAMMER kinase LkhA plays multiple roles in the vegetative growth and asexual and sexual development of Aspergillus nidulans. PLoS One 8:e58762. doi:10.1371/journal.pone.0058762. 23516554 PMC3596290

[55] Choi YK, Kang E-H, Park H-M 2014 Role of LAMMER kinase in cell wall biogenesis during vegetative growth of Aspergillus nidulans. Mycobiology 42:422–426. doi:10.5941/MYCO.2014.42.4.422. 25606019 PMC4298851

[56] Siebel CW, Feng L, Guthrie C, Fu XD 1999 Conservation in budding yeast of a kinase specific for SR splicing factors. Proc Natl Acad Sci U S A 96:5440–5445. doi:10.1073/pnas.96.10.5440. 10318902 PMC21878

[57] Taylor BL, Zhulin IB 1999 PAS domains: internal sensors of oxygen, redox potential, and light. Microbiol Mol Biol Rev 63:479–506. 10357859 10.1128/mmbr.63.2.479-506.1999 PMC98974

[58] Ho Y-SJ, Burden LM, Hurley JH 2000 Structure of the GAF domain, a ubiquitous signaling motif and a new class of cyclic GMP receptor. EMBO J 19:5288–5299. doi:10.1093/emboj/19.20.5288. 11032796 PMC314001

[59] Thomason P, Kay R 2000 Eukaryotic signal transduction via histidine-aspartate phosphorelay. J Cell Sci 113:3141–3150. 10954413 10.1242/jcs.113.18.3141

[60] Krems B, Charizanis C, Entian KD 1996 The response regulator-like protein Pos9/Skn7 of Saccharomyces cerevisiae is involved in oxidative stress resistance. Curr Genet 29:327–334. doi:10.1007/BF02208613. 8598053

[61] Posas F, Saito H 1998 Activation of the yeast SSK2 MAP kinase kinase kinase by the SSK1 two-component response regulator. EMBO J 17:1385–1394. doi:10.1093/emboj/17.5.1385. 9482735 PMC1170486

[62] Mulet JM, Leube MP, Kron SJ, Rios G, Fink GR, Serrano R 1999 A novel mechanism of ion homeostasis and salt tolerance in yeast: the Hal4 and Hal5 protein kinases modulate the Trk1-Trk2 potassium transporter. Mol Cell Biol 19:3328–3337. doi:10.1128/MCB.19.5.3328. 10207057 PMC84126

[63] Forment J, Mulet JM, Vicente O, Serrano R 2002 The yeast SR protein kinase Sky1p modulates salt tolerance, membrane potential and the Trk1,2 potassium transporter. Biochim Biophys Acta 1565:36–40. doi:10.1016/S0005-2736(02)00503-5. 12225850

[64] Stajich JE, Wilke SK, Ahrén D, Au CH, Birren BW, Borodovsky M, Burns C, Canbäck B, Casselton LA, Cheng CK, Deng J, Dietrich FS, Fargo DC, Farman ML, Gathman AC, Goldberg J, Guigó R, Hoegger PJ, Hooker JB, Huggins A, James TY, Kamada T, Kilaru S, Kodira C, Kües U, Kupfer D, Kwan HS, Lomsadze A, Li W, Lilly WW, Ma LJ, Mackey AJ, Manning G, Martin F, Muraguchi H, Natvig DO, Palmerini H, Ramesh MA, Rehmeyer CJ, Roe BA, Shenoy N, Stanke M, Ter-Hovhannisyan V, Tunlid A, Velagapudi R, Vision TJ, Zeng Q, Zolan ME, Pukkila PJ 2010 Insights into evolution of multicellular fungi from the assembled chromosomes of the mushroom Coprinopsis cinerea (Coprinus cinereus). Proc Natl Acad Sci U S A 107:11889–11894. doi:10.1073/pnas.1003391107. 20547848 PMC2900686

[65] Mascher T, Helmann JD, Unden G 2006 Stimulus perception in bacterial signal-transducing histidine kinases. Microbiol Mol Biol Rev 70:910–938. doi:10.1128/MMBR.00020-06. 17158704 PMC1698512

[66] Shor E, Chauhan N 2015 A case for two-component signaling systems as antifungal drug targets. PLoS Pathog 11:e1004632. doi:10.1371/journal.ppat.1004632. 25723524 PMC4344368

[67] Morgan DO 1995 Principles of CDK regulation. Nature 374:131–134. doi:10.1038/374131a0. 7877684

[68] Tamura K, Stecher G, Peterson D, Filipski A, Kumar S 2013 MEGA6: Molecular Evolutionary Genetics Analysis version 6.0. Mol Biol Evol 30:2725–2729. doi:10.1093/molbev/mst197. 24132122 PMC3840312

[69] Alikhan N-F, Petty NK, Ben Zakour NL, Beatson SA 2011 BLAST ring image generator (BRIG): simple prokaryote genome comparisons. BMC Genomics 12:402. doi:10.1186/1471-2164-12-402. 21824423 PMC3163573

[70] Bolger AM, Lohse M, Usadel B 2014 Trimmomatic: a flexible trimmer for Illumina sequence data. Bioinformatics 30:2114–2120. doi:10.1093/bioinformatics/btu170. 24695404 PMC4103590

[71] Liao Y, Smyth GK, Shi W 2013 The Subread aligner: fast, accurate and scalable read mapping by seed-and-vote. Nucleic Acids Res 41:e108. doi:10.1093/nar/gkt214. 23558742 PMC3664803

[72] Ritchie ME, Phipson B, Wu D, Hu Y, Law CW, Shi W, Smyth GK 2015 Limma powers differential expression analyses for RNA-sequencing and microarray studies. Nucleic Acids Res 43:e47. doi:10.1093/nar/gkv007. 25605792 PMC4402510

[73] Robinson MD, McCarthy DJ, Smyth GK 2010 edgeR: a Bioconductor package for differential expression analysis of digital gene expression data. Bioinformatics 26:139–140. doi:10.1093/bioinformatics/btp616. 19910308 PMC2796818

[74] Leslie JF, Summerell BA (ed) 2006 The *Fusarium* laboratory manual, p i–xii. Blackwell Publishing, Hoboken, NJ.

