# Supplementary figures and images for: Kinome Expansion in the Fusarium oxysporum Species Complex Driven by Accessory Chromosomes
Source: mSphere. 2018 Jun 13;3(3):e00231-18. doi: 10.1128/mSphere.00231-18 (PMC6001611; doi:10.1128/mSphere.00231-18)

a

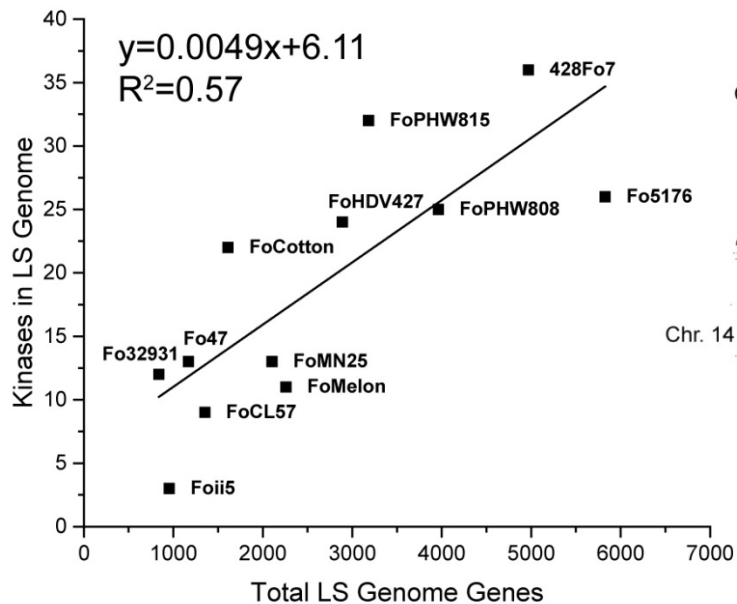

b

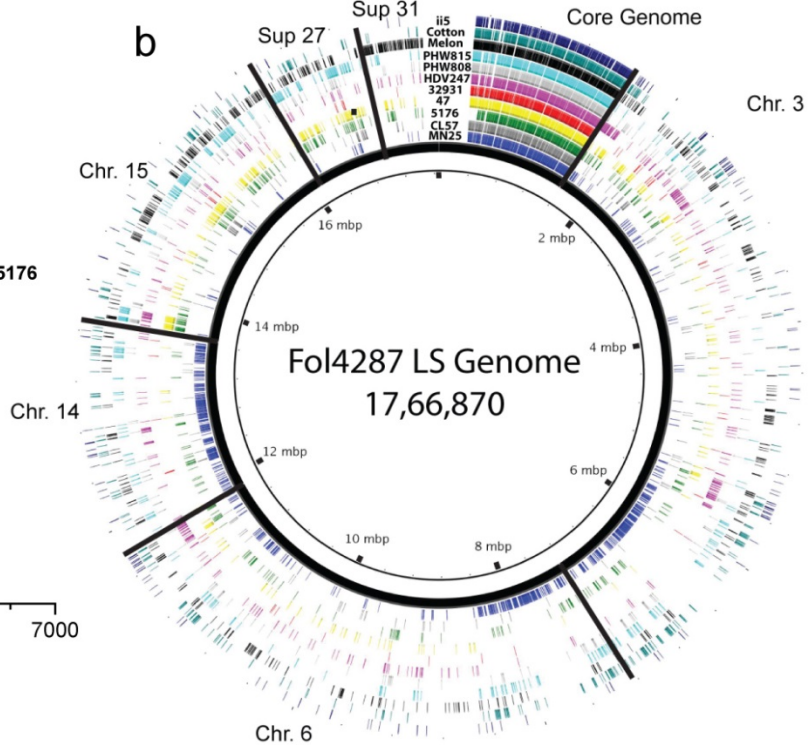

Supplement: FIG S1 [file sph003182566sf1.pdf]

a

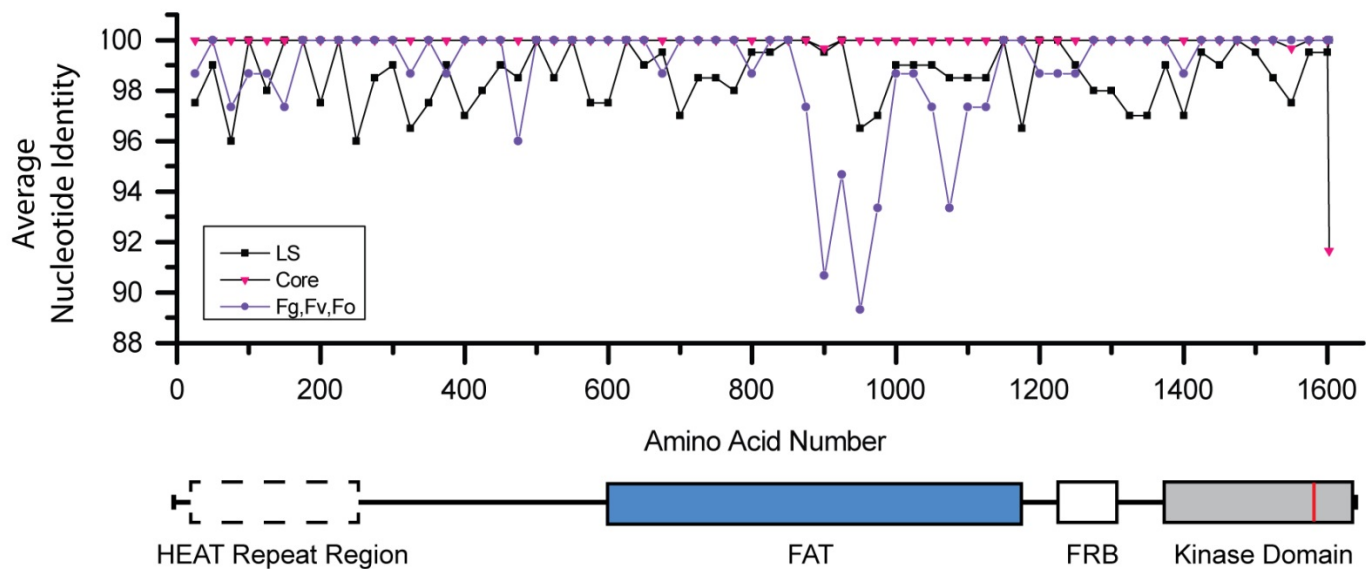

b

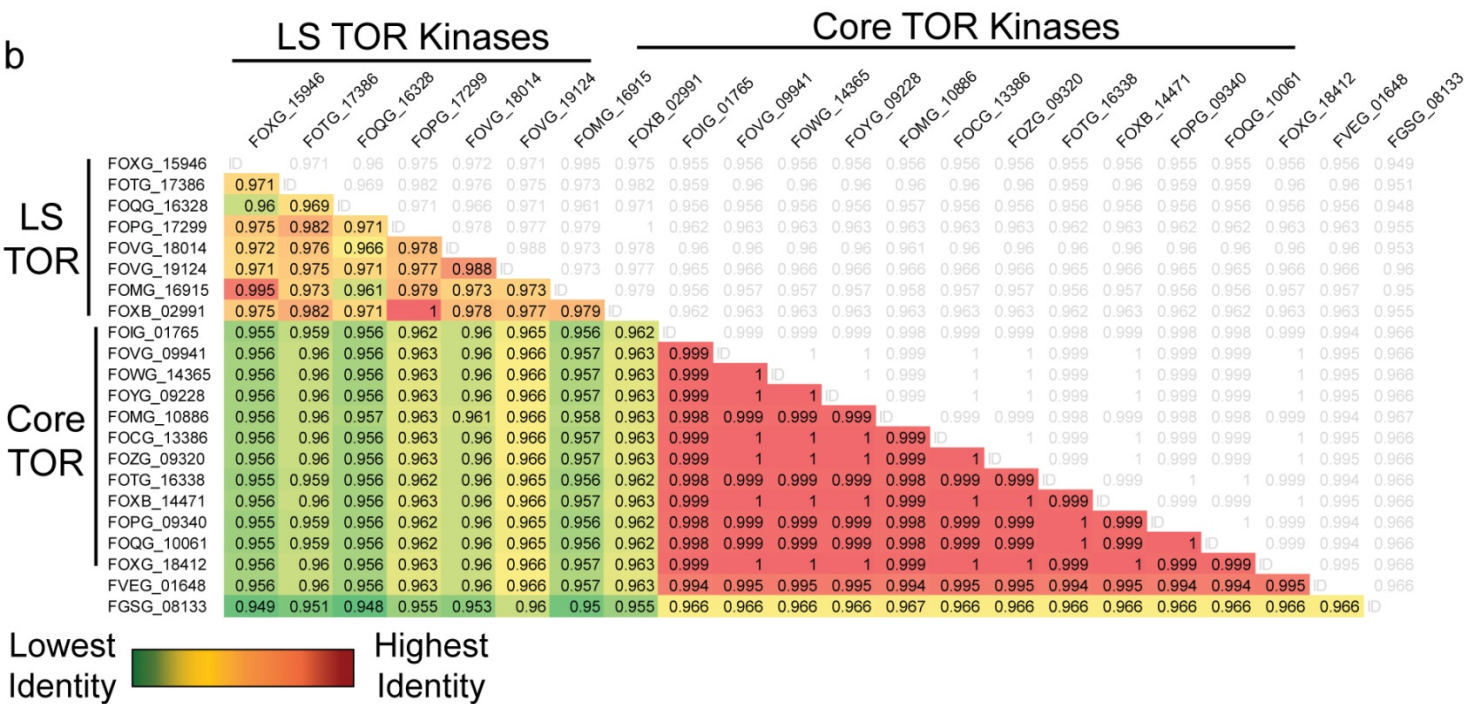

Supplement: FIG S2 [file sph003182566sf2.pdf]

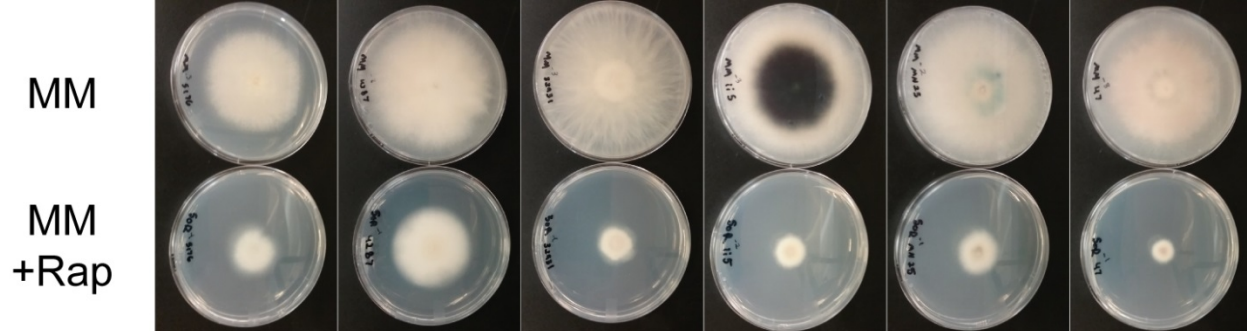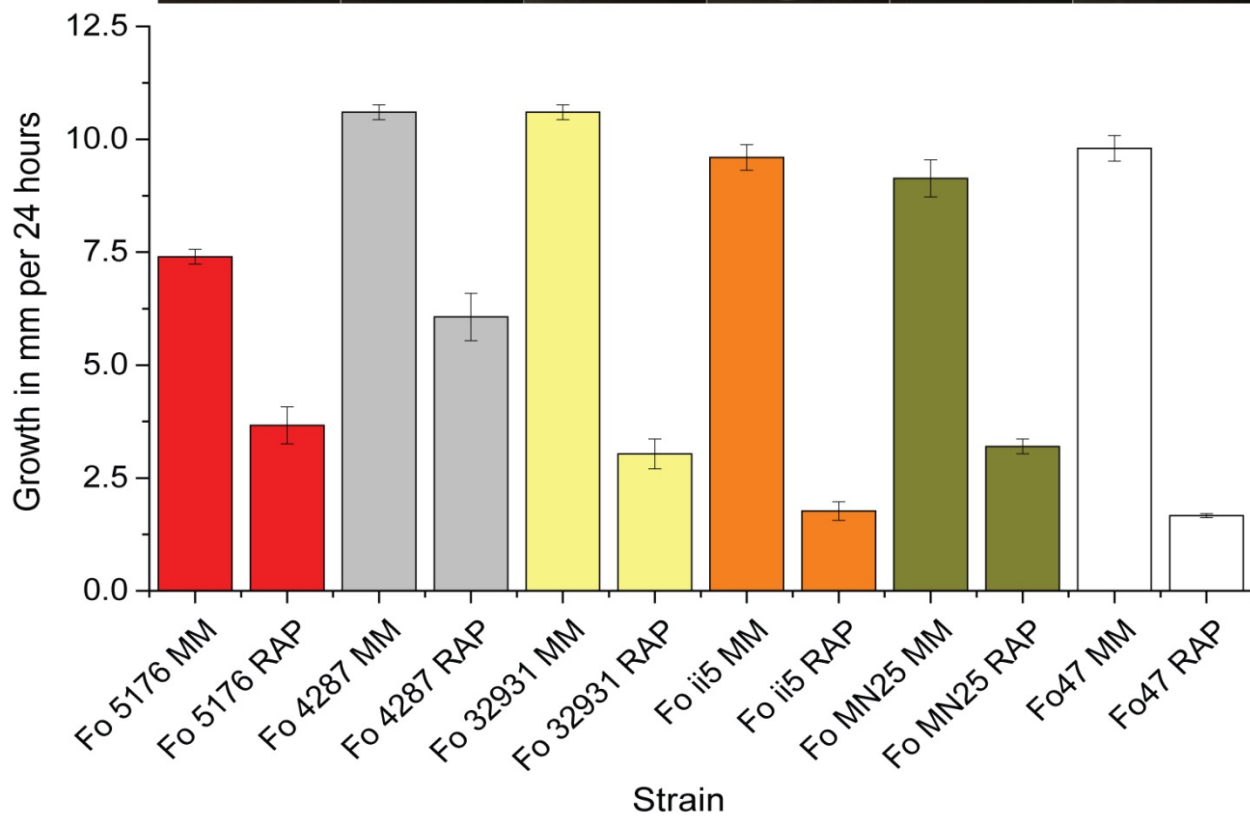

Supplement: FIG S3 [file sph003182566sf3.pdf]

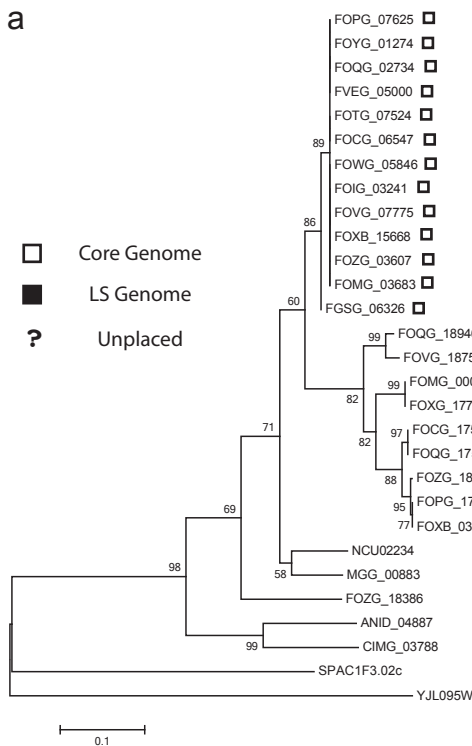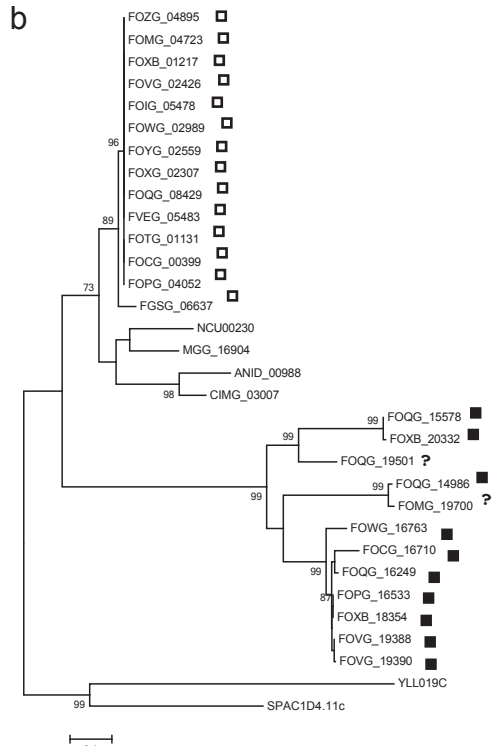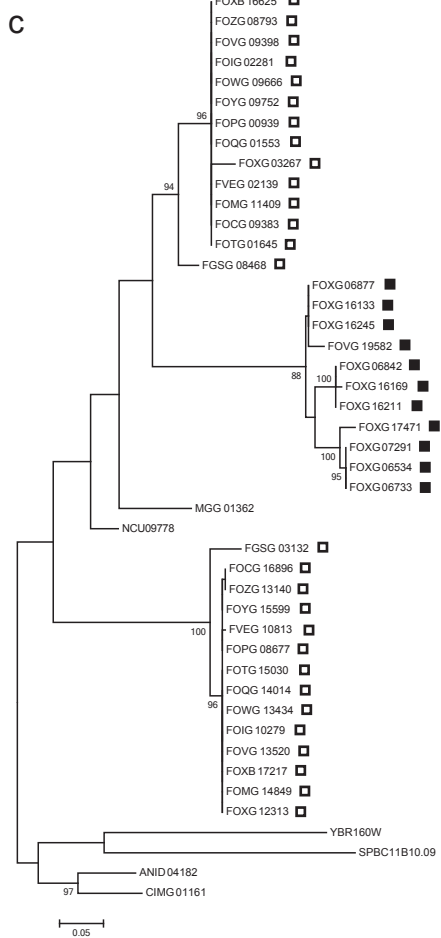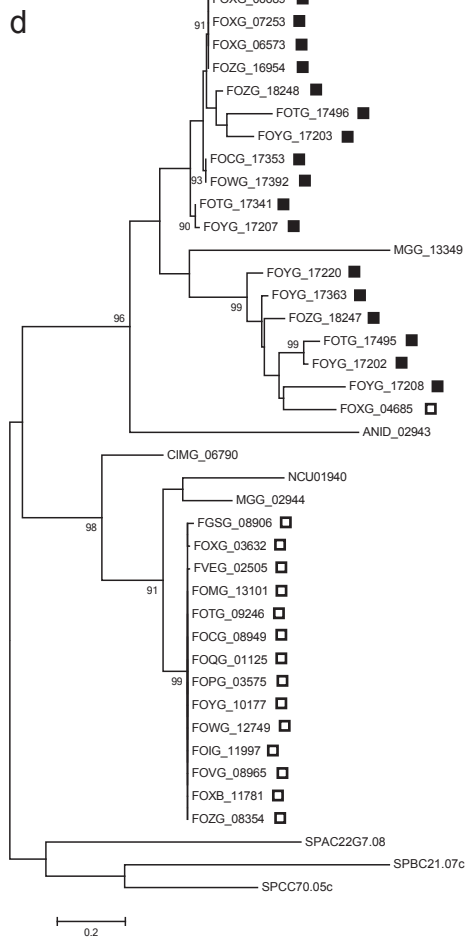

Supplement: FIG S4 [file sph003182566sf4.pdf]

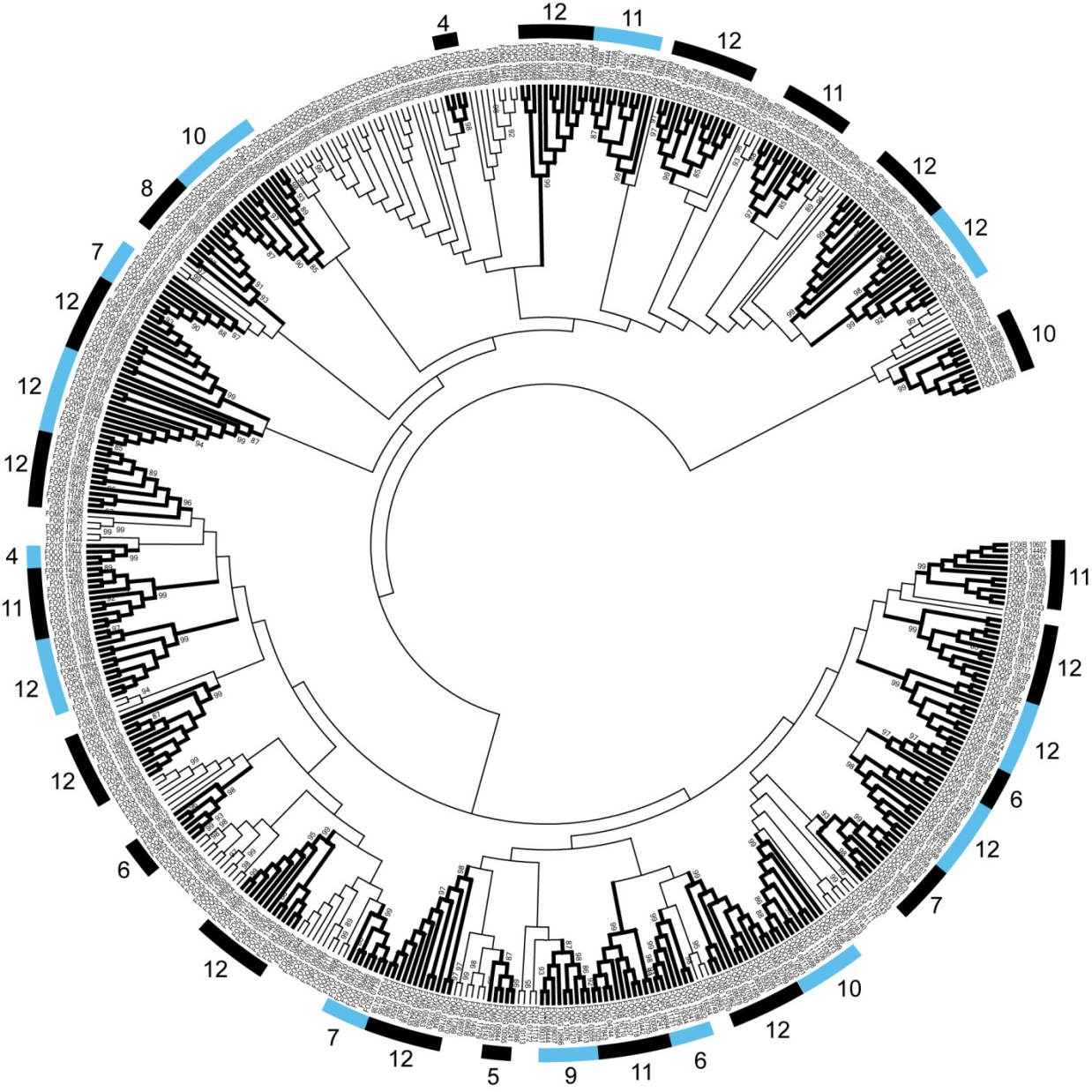

Supplement: FIG S5 [file sph003182566sf5.pdf]
